# Optimizing Plasmonic Gold Nanorod Deposition on Glass Surfaces for High-Sensitivity Refractometric Biosensing

**DOI:** 10.3390/nano12193432

**Published:** 2022-09-30

**Authors:** Youngkyu Hwang, Dong Jun Koo, Abdul Rahim Ferhan, Tun Naw Sut, Bo Kyeong Yoon, Nam-Joon Cho, Joshua A. Jackman

**Affiliations:** 1 School of Chemical Engineering and Translational Nanobioscience Research Center, Sungkyunkwan University, Suwon 16419, Korea; 2 School of Materials Science and Engineering, Nanyang Technological University, 50 Nanyang Avenue, Singapore 639798, Singapore; 3 School of Healthcare and Biomedical Engineering, Chonnam National University, Yeosu 59626, Korea

**Keywords:** nanoplasmonics, gold nanorod, localized surface plasmon resonance, self-assembled monolayer, biosensing

## Abstract

Owing to high surface sensitivity, gold nanorods (AuNRs) are widely used to construct surface-based nanoplasmonic biosensing platforms for label-free molecular diagnostic applications. A key fabrication step involves controlling AuNR deposition onto the target surface, which requires maximizing surface density while minimizing inter-particle aggregation, and is often achieved by surface functionalization with a self-assembled monolayer (SAM) prior to AuNR deposition. To date, existing studies have typically used a fixed concentration of SAM-forming organic molecules (0.2−10% *v*/*v*) while understanding how SAM density affects AuNR deposition and resulting sensing performance would be advantageous. Herein, we systematically investigated how controlling the (3-aminopropyl)triethoxysilane (APTES) concentration (1–30% *v*/*v*) during SAM preparation affects the fabrication of AuNR-coated glass surfaces for nanoplasmonic biosensing applications. Using scanning electron microscopy (SEM) and UV-visible spectroscopy, we identified an intermediate APTES concentration range that yielded the highest density of individually deposited AuNRs with minimal aggregation and also the highest peak wavelength in aqueous solution. Bulk refractive index sensitivity measurements indicated that the AuNR configuration had a strong effect on the sensing performance, and the corresponding wavelength-shift responses ranged from 125 to 290 nm per refractive index unit (RIU) depending on the APTES concentration used. Biosensing experiments involving protein detection and antigen–antibody interactions further demonstrated the high surface sensitivity of the optimized AuNR platform, especially in the low protein concentration range where the measurement shift was ~8-fold higher than that obtained with previously used sensing platforms.

## 1. Introduction

The interaction of light with plasmonic gold nanostructures such as nanoparticles, nanorods, nanodisks, and nanoislands can induce a wide range of optical phenomena stemming from the coherent oscillation of conduction-band electrons near the metal surface [1,2,3,4,5,6,7,8,9]. Among the different phenomena, localized surface plasmon resonance (LSPR) is one of the most promising ones for bioanalytical sensing applications and relates to a highly enhanced electromagnetic field near the sensing interface, which can be highly sensitive to changes in the local refractive index (e.g., due to contacting bio-analytes) [5,10,11,12,13,14,15]. Accordingly, the design of the nanostructure interface is critical to sensing performance and there are various methods to fabricate gold nanostructures on a solid surface. For example, lithographic methods (i.e., electron-beam lithography, focused ion beam lithography, and soft lithography) and coating methods based on chemical modification and templating (i.e., self-assembled monolayers and block copolymers) are among the possible options [16,17,18,19,20,21,22,23,24,25,26]. The latter case of depositing solution-phase gold nanostructures with defined size, shape, and composition onto a functionalized surface is particularly advantageous when considering the feasibility for mass production, low energy consumption, and high process efficiency [27,28,29,30]. 

Among different nanostructure types, gold nanorods (AuNRs) have received extensive attention due to unique optical properties that arise from their asymmetrical structure [26,31,32,33,34]. Specifically, AuNRs have two light absorption bands that are parallel and perpendicular to the long rod axis and are referred to as the transverse LSPR (t-LSPR, ~520 nm) and longitudinal LSPR (l-LSPR, ~650–1350 nm) peaks, respectively. The l-LSPR peak is known to have particularly high sensitivity to changes in the surrounding dielectric environment and this sensitivity typically becomes higher as the AuNR aspect ratio increases, which can be readily controlled using solution-phase synthesis. Hence, the controlled deposition of AuNRs on solid surfaces is an emerging strategy to develop plasmonic biosensors for label-free measurements [5,35,36,37,38].

To immobilize AuNRs on a surface, diverse material engineering strategies, including templates, oxygen plasma treatment, and self-assembled monolayers (SAMs), have been utilized to create nanostructured arrays for surface-enhanced Raman spectroscopy and metal-enhanced fluorescence applications [11,39,40,41]. In the case of SAM treatment, the first step involves the preparation of a SAM-functionalized surface using organic molecules that possess amino or thiol groups, for example, followed by deposition of AuNRs onto the functionalized surface. Depending on the specific protocol of the SAM coating step, there can be variations in the surface coverage and density of SAM molecules that impact resulting AuNR organization and density, which can in turn affect the plasmonic sensing performance as well. Interestingly, until now, the relevant studies [42,43,44,45,46,47,48,49,50] have used various concentrations of SAM-forming organic molecules (typically 0.2–10% *v*/*v*) and incubation times to prepare the SAM-functionalized surface, while it would be advantageous to investigate how optimizing the SAM functionalization step can modulate AuNR deposition and in turn influence refractometric biosensing performance (Table S1). The latter aspect is especially important not only in terms of bulk refractive index sensitivity, but also in terms of molecular surface sensitivity and highlights how a nanoarchitectonics design approach can be useful to improve the sensing performance of deposited AuNR arrays for biosensing applications.

Towards this goal, herein, we investigated how controlling AuNR organization and density on a SAM-functionalized glass surface influences plasmonic biosensing performance in terms of maximizing bulk refractive index sensitivity and detection sensitivity for tracking noncovalent protein adsorption and multistep antibody–antigen interactions. Central to our approach was the use of (3-aminopropyl)triethoxysilane (APTES), which is an organosilane molecule with an amino terminal group, that was used to form the SAM-functionalized glass surface, and the APTES concentration was systematically varied to modulate the AuNR distribution, density, and aggregation, all of which impact refractometric biosensing performance. Indeed, while achieving a high AuNR density is useful for improved sensitivity, it can also lead to AuNR aggregation that affects the plasmonic signal readout, and hence, optimizing the AuNR platform coating properties is important. Following this approach, we characterized the density and organization of deposited AuNRs using scanning electron microscopy (SEM), followed by spectroscopic characterization of the light extinction spectra and biosensing performance. Our findings provide insight into effective surface engineering, for example, optimizing SAM treatment conditions for AuNR deposition that can boost plasmonic sensing performance and can be broadly applicable to plasmonic nanostructures in general. Specifically, our study provides the first systematic investigation unravelling how AuNR surface density and attachment-related nanorod aggregation influence nanoplasmonic sensing properties in terms of bulk refractive index sensitivity and molecular surface sensitivity, whereby optimizing the APTES concentration based on the insights obtained in this study significantly improved both of these sensing parameters. 

## 2. Materials and Methods

### 2.1. Fabrication of AuNR-Coated Glass Substrate

Glass substrates (Fisherbrand^TM^ Superfrost^TM^ Plus, Thermo Fisher Scientific, Waltham, MA, USA) were cleaned with deionized water (MilliporeSigma, Burlington, MA, USA), and treated with oxygen plasma for 5 min by using a CUTE-1MPR machine (Femto Science Inc., Hwaseong, Korea). The samples were incubated in an ethanolic solution containing different concentrations (1–30% *v*/*v*) of (3-aminopropyl)triethoxysilane (APTES) (MilliporeSigma, Burlington, MA, USA) for 30 min under shaking at 50 rpm and left for 30 min without shaking. After APTES incubation, they were rinsed with ethanol, followed by blowing with N_2_ gas and then baked in an oven at 110 °C for 60 min. Next, the samples were incubated in an AuNR solution (Sigma-Aldrich, St. Louis, MO, USA; 40 µg/mL, dimensions: 10 nm × 37 nm) in a dark environment for 24 h. After the coating process, the samples were gently washed with deionized water and dried with N_2_ gas.

### 2.2. Substrate Characterization

SEM images were obtained using a JSM-7600F Schottky field-emission scanning electron microscope (JEOL, Tokyo, Japan). The number density of deposited AuNRs in the SEM images was analyzed by ImageJ software (National Institutes of Health, Bethesda, MD, USA). Optical extinction spectra were recorded using a microplate reader (SpectraMax iD5, Molecular Devices, San Jose, CA, USA). Bulk refractive index sensitivity values were measured by incubating the AuNR-coated substrates in water–glycerol mixtures with increasing glycerol fractions (0–40% *v*/*v*). To measure the bulk RI sensitivity, the maximum intensity wavelength (Δλ_max_) was obtained as a function of the change in refractive index units (ΔRIU) for different water–glycerol mixtures and the slope (Δλ_max_/ΔRIU) was calculated by linear regression analysis [51]. 

### 2.3. Protein Detection Measurements

Samples with different concentrations (0.001–100 μM) of bovine serum albumin (BSA) protein (MilliporeSigma, Burlington, MA, USA) were prepared in 10 mM Tris buffer (150 mM NaCl, pH 7.5), were added to the AuNR-coated substrates for 30 min under shaking at 50 rpm and then washed three times with Tris buffer. The optical extinction spectrum was measured at each step by a microplate reader. A 500 μL aliquot of a 5 µg/mL recombinant COVID-19 nucleocapsid (N) antigen (catalog no. LIC-NP-04, Luca AICell, Inc., Anyang, Korea) in a carbonate coating buffer (50 mM, pH 9.6) was added to the AuNR-coated glass surface in the plate wells and incubated at 37 °C for 1 h. Then, the wells were washed 3 times with 1 mL of PBS with 0.05% Tween 20 (PBS-T). After washing, 1 mL volume of monoclonal primary antibody (clone 12D12, Luca AICell, Inc.) in PBS-T was incubated with the antigen-coated surface in the wells at 37 °C for 1 h, followed by washing 3 times with PBS-T. Then, 500 μL of the secondary antibody (1:20,000 dilution, Luca AICell, Inc.) in PBS-T was added to the surface in the wells and then incubated at 37 °C for 1 h. After washing 3 times with PBS-T, the optical extinction spectrum of the AuNR-coated surface after respective incubation steps with the antigen, primary and second antibody, were measured by using a microplate reader. All measurements were conducted at room temperature (~24 °C). In applicable cases, additional morphological characterization was performed by atomic force microscopy (AFM) experiments with a JPK NanoWizard Ultra Speed instrument operated in non-contact mode (Bruker Nano GmbH, Berlin, Germany). In addition, Fourier transform infrared spectroscopy (FTIR) experiments were conducted using an FT-IR 4700 spectrometer (JASCO, Tokyo, Japan) with an attenuated total reflectance (ATR) accessory module. Sample spectra were obtained between 4000 and 650 cm^–1^ with 32-times scanning per measurement by using single-reflection ATR mode (incident light angle: 45°). Background spectra collected prior to sample readings were subtracted from sample spectra, and a baseline correction process was also performed. 

## 3. Results

**Figure 1:**
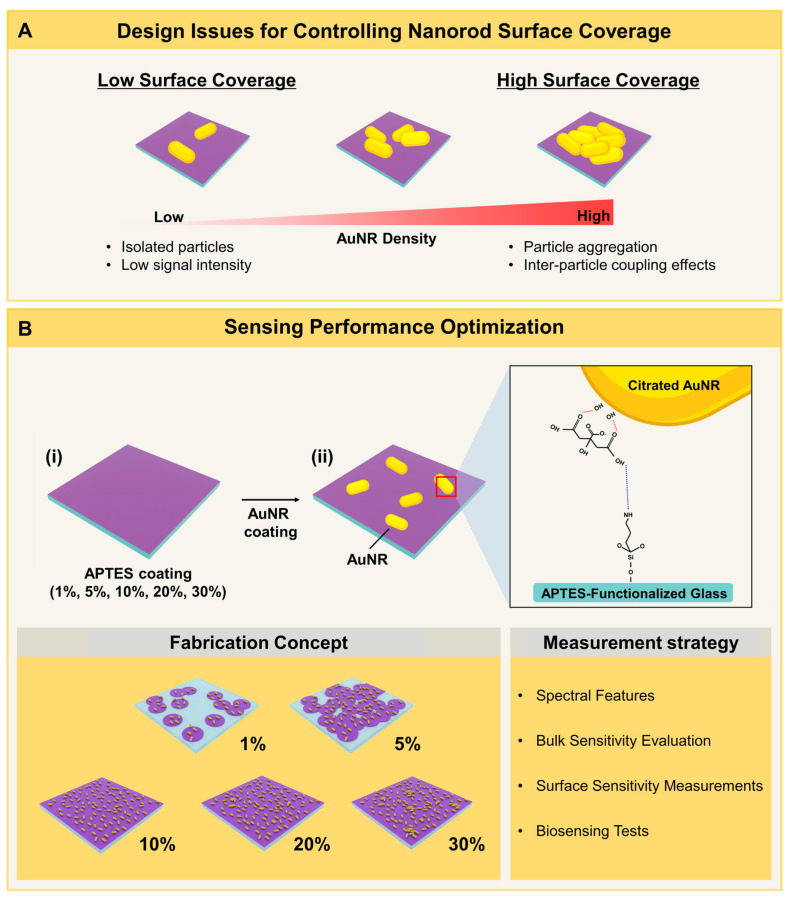
(**A**) Overview of technical considerations for preparing low- and high-coverage AuNR sensing platforms. (**B**) Fabrication concept and measurement strategy to control AuNR platform properties by varying the APTES concentration.

We begin by introducing the key design objectives to consider when fabricating APTES-functionalized surfaces based on the deposition of solution-phase nanostructures such as AuNRs. Figure 1A presents the major issues to consider when deposited AuNRs have different surface coverages. At low coverage, the AuNRs are well-separated, but the low density can decrease plasmonic signal intensity. On the other hand, at high coverage, there can be AuNR aggregation and the resulting inter-particle coupling effects can cause distortions in the plasmonic signal readout, especially for refractometric biosensing applications. To address this issue, we prepared APTES-functionalized glass surfaces using different APTES concentrations (1–30% *v*/*v*, positive charge) and citrated AuNRs (negative charge) were attached to the APTES moieties via electrostatic attraction (Figure 1B and Figure S1). We hypothesized that controlling the APTES coating conditions would in turn affect the surface coverage of deposited AuNRs and performed various plasmonic biosensing experiments to identify optimal platform conditions.

Briefly, the coating process started with washing a glass substrate with acetone and deionized (DI) water under sonication for 5 min and then blowing the surface using a N_2_ gas stream to remove residual DI water. After O_2_ plasma treatment for 5 min to generate hydroxyl groups on the glass surface, various concentrations of ethanolic APTES solution were incubated with the substrate under orbital shaking for 30 min and then left for 30 min without shaking. After washing the samples with ethanol followed by treatment with a N_2_ gas stream, the samples were incubated in an oven at 110 °C for 60 min.

The APTES-functionalized glass surface was incubated with AuNRs in DI water (250 µL, density: 35 µg/mL, length: 37 ± 7 nm, diameter: 10 ± 2 nm) under orbital shaking for 30 min and then left overnight (for 23.5 h) without shaking, followed by rinsing with aqueous solution to remove weakly attached AuNRs. 

**Figure 2:**
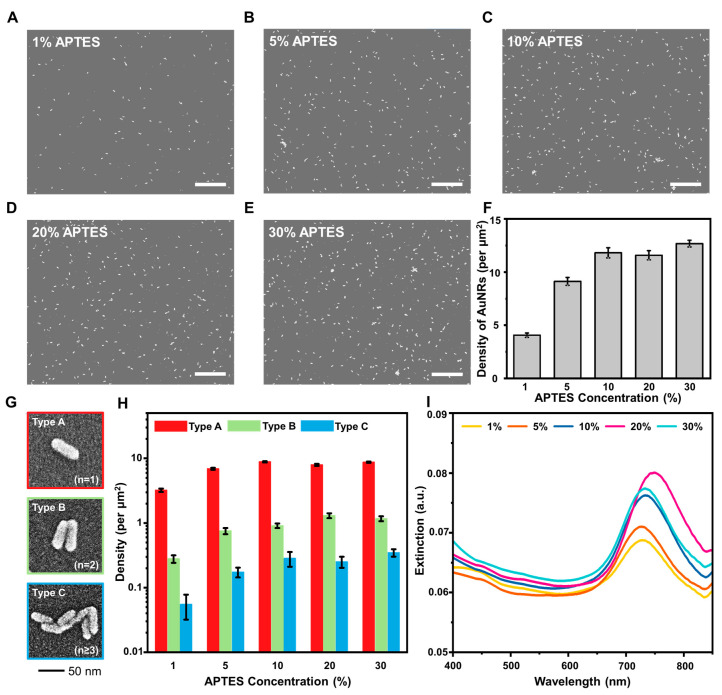
(**A**–**E**) SEM images of deposited AuNRs on glass substrate as a function of the APTES concentration (1–30% *v*/*v* in ethanol) used during fabrication. Scale bars are 1 µm. (**F**) Surface density of deposited AuNRs on the glass substrate as a function of APTES concentration and expressed in terms of nanorods per µm^2^. (**G**) Representative SEM images corresponding to deposited AuNRs in different aggregation states that are defined as Type A (individual AuNR), Type B (two AuNRs adjoined together), and Type C (three or more AuNRs adjoined together). (**H**) Surface density of deposited AuNRs in the different aggregation states based on the data in panel (**G**). (**I**) Optical extinction spectra of deposited AuNRs on glass substrate as a function of the APTES concentration. Data in panels (**F**) and (**H**) are reported as the mean ± standard deviation from three experiments.

To confirm the effect of APTES concentration on AuNR attachment density, we characterized the fabricated AuNR arrays by SEM imaging (Figure 2A–E). The number density of AuNRs on the surface noticeably increased as the APTES concentration used was increased up to 10%. For the 1%, 5%, and 10% APTES cases, the AuNR surface density was 4.0 ± 0.2 µm^−2^, 9.0 ± 0.4 µm^−2^, and 11.9 ± 0.5 µm^−2^, respectively (Figure 2F). On the other hand, for the 20% and 30% APTES cases, there was no significant increase in the AuNR number density compared to the 10% APTES case. In all cases, there was still a relatively low surface density of attached AuNRs, which is suitable for LSPR-based sensing applications where it is ideal to minimize inter-particle coupling [51,52].

In addition to total number density, we also analyzed the SEM images at higher magnification to investigate how the APTES concentration used during the coating step in turn affected the aggregation state of deposited AuNRs on the functionalized glass surface (Figure 2G). We classified the deposited AuNRs in terms of three aggregation states: Type A consisted of individual AuNRs; Type B consisted of two AuNRs joined together; and Type C consisted of three or more AuNRs joined together. The surface density of AuNRs (nanorods per µm) in the Type A configuration ranged from 3.2 ± 0.2 µm^−2^ in the 1% APTES case to 8.8 ± 0.2 µm^−2^ in the 10% APTES case, and similarly high values in the 20% and 30% APTES cases indicate that saturation was reached (Figure 2H). On the other hand, the surface density of AuNRs in the Type B and C configurations also tended to increase at higher APTES concentrations and hence the use of 10–20% APTES concentration for substrate fabrication appeared to be optimal in terms of maximizing the surface density of isolated AuNRs while minimizing the presence of AuNR aggregates.

We proceeded to characterize the plasmonic properties of the AuNR platforms by measuring the optical extinction spectra (Figure 2I). As the APTES concentration used was varied from 1% to 20%, the highest-intensity peak wavelength in the extinction spectrum showed a red shift from 729 nm to 750 nm, which is associated with an increase in the surface density of individual AuNRs and a corresponding decrease in inter-particle gap distance. On the other hand, for the 30% APTES case, a blue shift occurred that is related to randomly arranged AuNR aggregates with decreased anisotropy and hence lower sensitivity [31,53,54,55,56,57,58].

**Figure 3:**
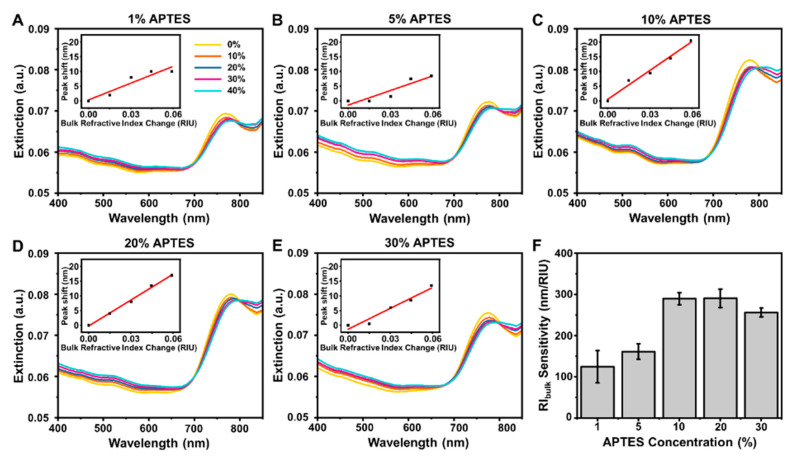
(**A**–**E**) Representative optical extinction spectra of fabricated AuNR platforms in different water-glycerol mixture environments (0–40% *v*/*v* glycerol). Insets are linear fits of the corresponding Δλ_max_ shifts as a function of the bulk refractive index change in the different water–glycerol mixtures. The platforms were fabricated using different APTES concentrations to functionalize the glass surface prior to AuNR deposition. (**F**) Summary of bulk refractive index values for different AuNR platforms. Data are reported as the mean ± standard deviation from three experiments.

We also performed refractive index (RI) sensitivity measurements on the different AuNR platforms by tracking the change in the l-LSPR peak wavelength (Δλ_max_) in the presence of different water–glycerol mixtures (0–40%, in 10% increments). This measurement approach is well-established for calibration and for determining the bulk RI sensitivity value, which is an important sensing performance metric [59]. With increasing glycerol fraction, the mixtures had larger refractive index unit (RIU) values and the first measurement step was done in water, before exchanging the bulk solution with mixtures that contained increasingly larger glycerol fractions. Based on this approach, the Δλ_max_ shift per ΔRIU shift was calculated from linear slope analysis and is defined as the bulk RI sensitivity value. In the 1% and 5% APTES cases, the AuNR-coated glass substrates had low bulk RI sensitivities of around 124.6 ± 39.0 and 161.5 ± 18.5 nm/RIU, respectively (Figure 3A,B). Of note, there was also relatively weak extinction intensity due to the low surface density of AuNRs on the substrate and poor linearity in the signal response. 

On the other hand, in the 10% and 20% APTES cases, there was much higher measurement sensitivity and the bulk RI sensitivity values in those cases were 289.5 ± 14.5 and 290.3 ± 22.5 nm/RIU, respectively, with good linearity (Figure 3C,D). By contrast, in the 30% APTES case, the bulk RI sensitivity decreased to 256.0 ± 10.7 nm/RIU and there was lower extinction intensity due to greater presence of AuNR aggregates (Figure 3E). Based on these results, 10–20% APTES was identified to be a suitable range for preparing highly sensitive AuNR platforms and we selected AuNR platforms prepared using 20% APTES concentration for further biosensing evaluation (Figure 3F). Selection of this particular AuNR platform was further reinforced by verifying its stability since there were negligible changes in the corresponding optical extinction spectrum upon extensive washing (for each cycle, three-times rinsing with water followed by nitrogen gas drying) (Figure S2).

**Figure 4:**
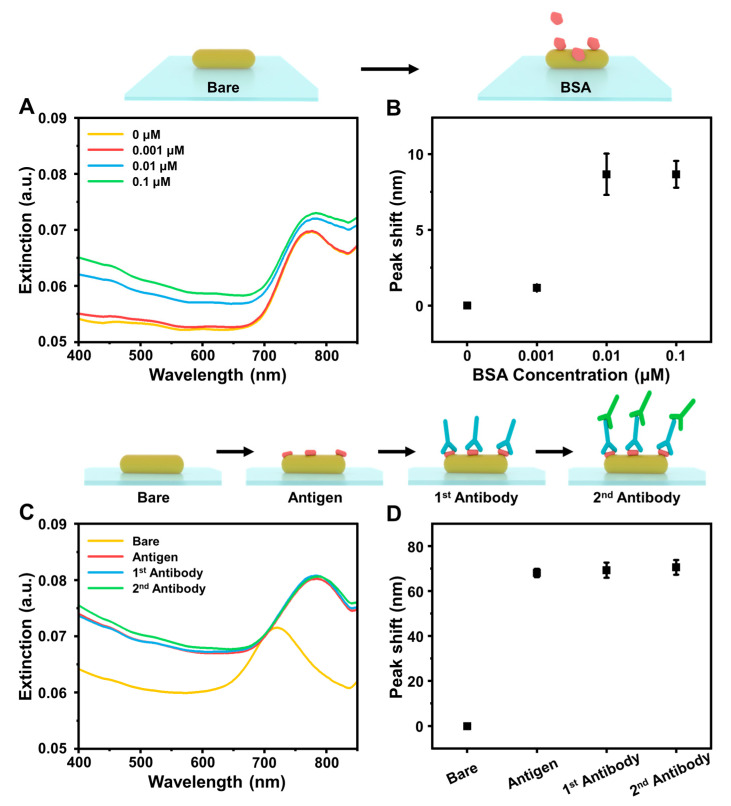
(**A**) Representative optical extinction spectra for BSA protein adsorption onto fabricated AuNR platform in the presence of different bulk protein concentrations. (**B**) Summary of final Δλ_max_ shifts corresponding to data in panel (**A**). (**C**) Representative optical extinction spectra for sequential addition of COVID-19 N antigen, primary antibody, and secondary antibody onto fabricated AuNR platform. (**D**) Summary of final Δλ_max_ shifts corresponding to data in panel (**C**). Data in panels (**B**) and (**D**) are reported as the mean ± standard deviation from three experiments.

In addition to bulk refractive index sensitivity, another key parameter is the molecular sensitivity to detect biomacromolecular interactions related to changes in the local refractive index near the sensor surface. This molecular sensitivity can be evaluated by using different-sized protein molecules, which can adsorb onto the sensor surface and form a thin-film adsorbate to measure corresponding measurement responses that relate to the LSPR probing volume, i.e., degree of surface sensitivity [60,61]. First, we measured the noncovalent adsorption of bovine serum albumin (BSA) protein onto the surface and observed a peak shift increase due to protein adsorption (Figure 4A). Quantitatively, the Δλ_max_ shift increased from 1.2 ± 0.2 nm for 0.001 μM BSA concentration to 8.7 ± 1.4 nm and 8.7 ± 0.9 nm for 0.01 μM and 0.1 μM BSA concentrations, respectively (Figure 4B). While most related studies have investigated BSA protein detection at ~10–100 μM concentrations, noteworthy points of these results are that similarly large measurement responses were recorded at appreciably lower concentrations using our platform and that the measurement response saturates at relatively low protein concentration in our case. These points emphasize that our sensing platform is particularly well suited for detecting protein analytes at very low concentrations, which could relate to protein–nanorod interactions and resulting protein conformational changes [62]. BSA adsorption to the AuNR platform was further characterized by atomic force microscopy (AFM) imaging, which indicated that the maximum height features increased from ~10 nm to ~15 nm due to BSA attachment (BSA molecules have typical height of ~5 nm) and the root-mean-square surface roughness of the surface also increased from 1.005 nm to 1.431 nm after BSA attachment [63,64,65]. In addition, Fourier transform infrared spectroscopy (FTIR) experiments verified BSA attachment, as indicated by a strong peak at 1654 cm^−1^ that corresponded to the amide I band of BSA protein molecules [66] (Figures S3 and S4).

Aside from BSA adsorption, we also measured the attachment of coronavirus disease-2019 (COVID-19) nucleocapsid (N) protein antigen and antibodies to the AuNR platform. In this case, the N antigen was initially added, and its adsorption onto the sensor surface resulted in a large Δλ_max_ shift of around 68.0 ± 1.8 nm (Figure 4C,D). On the other hand, after antigen attachment, subsequent addition of primary and secondary antibodies only led to Δλ_max_ shifts of around ~1.3 nm and ~2.5 nm, respectively, which is consistent with the large molecular size of these protein analytes (>100 kDa), the sensor surface being occupied by already-attached antigen molecules, and the short decay length of the LSPR-enhanced electromagnetic fields (~5–10 nm) when plasmonic nanoparticles are well separated and there is a low degree of inter-particle plasmon coupling [60,67]. In conjunction with the microplate reader, we further estimated that the sensor resolution was on the order of 3 × 10^−3^ RIU (3 × standard deviation of the Δλ_max_ baseline signal) while the AuNR platform can be readily integrated with dedicated UV-vis spectrophotometers to reach on the order of 10^-5^–10^-6^ RIU [68].

**Figure 5:**
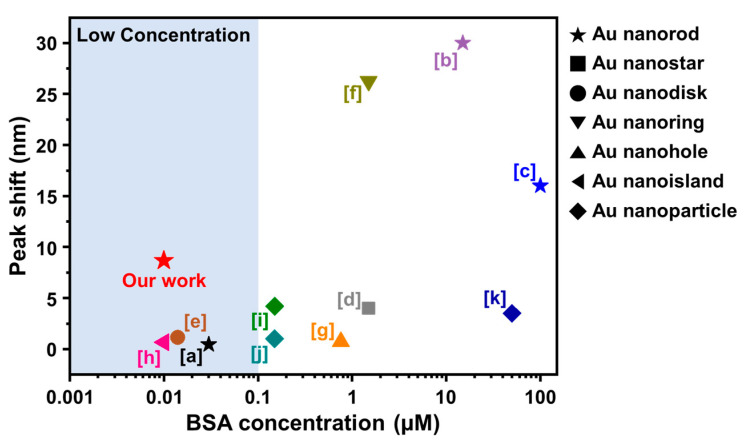
Comparison of nanoplasmonic biosensing performance of the AuNR platform in this study compared to other gold nanostructures reported in the literature. The data correspond to the reported LSPR Δλ_max_ shifts for BSA protein adsorption at different bulk protein concentrations. The literature results were obtained from the following references: a-[69], b-[70], c-[45], d-[71], e-[72], f-[73], g-[74], h-[9], i-[75], j-[76], and k-[77].

To contextualize these biosensing performance results, the reported LSPR-related peak shifts for BSA adsorption onto various plasmonic gold nanostructures are presented in Figure 5. 

While most studies have focused on relatively high BSA concentrations (>0.1 µM), our study demonstrates that AuNRs have strong merits for detecting protein analytes at lower concentrations as well. Within the low concentration regime (<0.1 µM), our AuNR platform demonstrated an ~8-times larger measurement response compared to other plasmonic nanostructures. Notably, a quite small measurement response of ~0.3 nm was recorded for 0.03 µM BSA to polymer-coated AuNRs [69], whereas the bare AuNR platform in this study had an ~8-nm peak shift for 0.01 µM BSA.

It should also be remarked that our study focused on the APTES-mediated attachment of AuNRs onto a functionalized glass surface, which results in a quasi-two-dimensional (2D) arrangement of AuNRs on the surface. This approach led us to determine that the nanoplasmonic sensing performance of this particular AuNR platform with a specific nanoparticle shape could be optimized by maximizing the surface density of attached AuNRs while minimizing the presence of AuNR aggregates on the surface, yielding an over ~2-fold improvement in bulk refractive index sensitivity up to 290 nm/RIU. Other AuNR platforms utilizing AuNRs with different shapes, i.e., larger major axes (which is known to be related to sensing performance; see Refs. [78,79]), have reported higher bulk refractive index sensitivities in excess of 400 nm/RIU, and it is anticipated that the general design principles identified in our study could be applied to those other AuNR platforms as well [80]. In addition, the development of three-dimensional (3D) arrangements of AuNRs embedded within soft-matter assemblies such as thin-film polyelectrolyte complexes might also consider these design principles in order to boost sensing performance [81].

## 4. Conclusions

In this study, we have systematically investigated the design and fabrication of AuNR-coated glass surfaces by varying the APTES concentration that was used to functionalize the glass surface prior to AuNR deposition. While the basic fabrication strategy is long established, there has been a wide range of APTES concentrations (0.2–10% *v*/*v*) used to functionalize the glass surface in prior studies, and our findings indicate that controlling this parameter can strongly influence the AuNR platform architecture in terms of surface density and particle aggregation, and resulting sensing properties. An intermediate APTES concentration in the range of 10–20% *v*/*v* was identified to be optimal for maximizing AuNR surface density, minimizing aggregation, and contributing to a high peak wavelength in aqueous solution. Notably, the bulk refractive index sensitivity of the AuNR platforms varied from 125 to 290 nm/RIU depending on the APTES concentration used during fabrication and was correlated with the nanoarchitecture properties of the deposited AuNRs. Together with the demonstrated high sensitivity to detect protein analytes, these results highlight how optimizing AuNR deposition can boost nanoplasmonic sensing performance based on a simple yet effective interfacial science strategy to maximize the density of individual AuNRs while minimizing aggregation, and such possibilities are potentially extendable to other types of biosensing devices such as liquid crystal sensors [82,83].

## Data Availability

Data are available from the authors upon request.

## Supplementary Materials

The following supporting information can be downloaded at: https://www.mdpi.com/article/10.3390/nano12193432/s1, Table S1. Comparison of gold nanostructure coating conditions in relevant studies, including APTES concentration, solvent, and incubation time; Figure S1: Photographs of AuNR platforms on APTES-coated glass surfaces depending on the APTES concentration that was used during fabrication; Figure S2: Optical extinction spectra of deposited AuNRs on a glass substrate after repeated washing cycles; Figure S3: Surface morphology of AuNR platform; Figure S4: FTIR spectroscopic analysis of glass surface without and with BSA protein coating and AuNR-coated glass surface without and with BSA protein coating. References [45,77,84,85,86,87,88,89,90,91,92,93,94] are cited in the Supplementary Materials.
