# Supplementary material for: Optimizing Plasmonic Gold Nanorod Deposition on Glass Surfaces for High-Sensitivity Refractometric Biosensing
Source: Nanomaterials (Basel). 2022 Sep 30;12(19):3432. doi: 10.3390/nano12193432 (PMC9565783; doi:10.3390/nano12193432)
Supplement: Supplementary file 1 [file nanomaterials-12-03432-s001.zip › nanomaterials-1864593-supplementary.pdf]

Supplementary Information

Article

Optimizing Plasmonic Gold Nanorod Deposition on Glass Surfaces for High-Sensitivity Refractometric Biosensing

Youngkyu Hwang [1,†], Dong Jun Koo [1,†], Abdul Rahim Ferhan [2], Tun Naw Sut [1], Bo Kyeong Yoon [3], Nam-Joon Cho [2,*] and Joshua A. Jackman [1,*]

[1] School of Chemical Engineering and Translational Nanobioscience Research Center, Sungkyunkwan University, Suwon 16419, Korea
[2] School of Materials Science and Engineering, Nanyang Technological University, 50 Nanyang Avenue, Singapore 639798, Singapore
[3] School of Healthcare and Biomedical Engineering, Chonnam National University, Yeosu 59626, Korea
* Correspondence: njcho@ntu.edu.sg (N.-J.C.); jjackman@skku.edu (J.A.J.)
† These authors contributed equally to this work.

Table S1. Comparison of gold nanostructure coating conditions in relevant studies, including APTES concentration, solvent, and incubation time.

APTES Conc. (%)	Solvent	Incubation (h)	Substrate	Type of Au nanostructure	Dimension (nm) diameter (D), height (H)	Ref.
1-30	EtOH	1	Glass	AuNR[b]	D: 10, H: 37	Our work
0.2	Toluene	2	Glass	AuNBP[c]	D: 35, H: 70	[1]
1	EtOH	6	PDMS	AuNP[d]	D: 100	[2]
1	EtOH	24	Glass	AuNP	D: 10–15	[3]
5	Water	0.33	Glass	AuNP	D: 13	[4]
5	IPA[a]	1.5	Glass	AuNP	D: 40	[5]
5	EtOH	2	Glass	AuNP	D: 15	[6]
5	EtOH	6	Silicon	AuNS[e]	D: 80	[7]
10	MeOH	1	Silicon	AuNP	D: 13	[8]
10	MeOH	2	Silicon	AuNP	D: 3	[9]
10	EtOH	0.25	Glass	AuNP	D: 13	[10]
10	EtOH	0.33	Glass	AuNP	D: 30	[11]
10	EtOH	0.5	Glass	AuNR	D: 20, H: 50	[12]
10	EtOH	0.5	Silicon	AuNP	D: 15	[13]

[a]IPA: Isopropyl alcohol; [b]AuNR: Au nanorod; [c]AuNBP: Au nanobipyramid; [d]AuNP: Au nanoparticle; [e]AuNS: Au nanostar

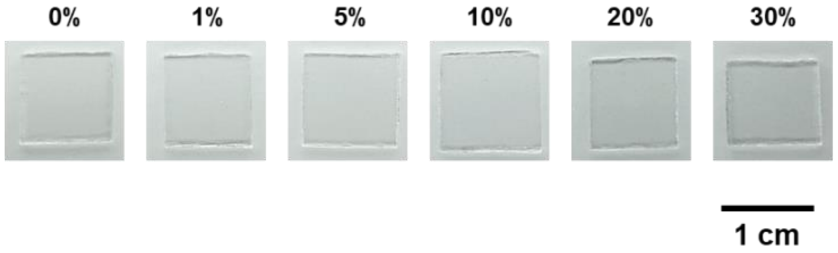

Figure S1. Photographs of AuNR platforms on APTES-coated glass surfaces depending on the APTES concentration that was used during fabrication.

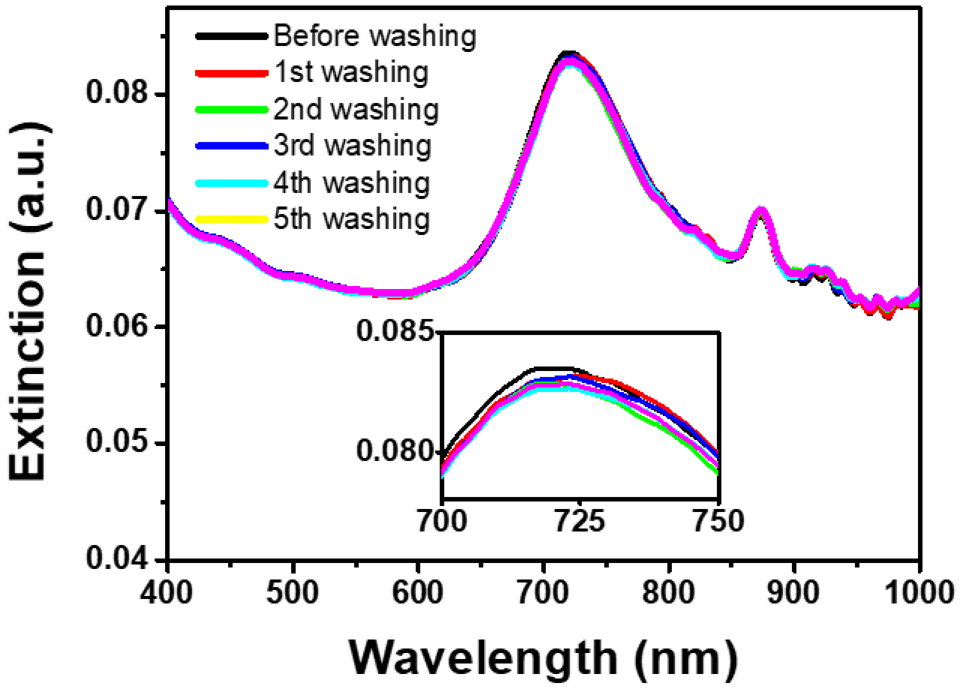

Figure S2. Optical extinction spectra of deposited AuNRs on a glass substrate after repeated washing cycles. The APTES concentration used to prepare the platform was 20% and each washing cycle consisted of three-times water rinsing and then drying with a nitrogen gas stream.

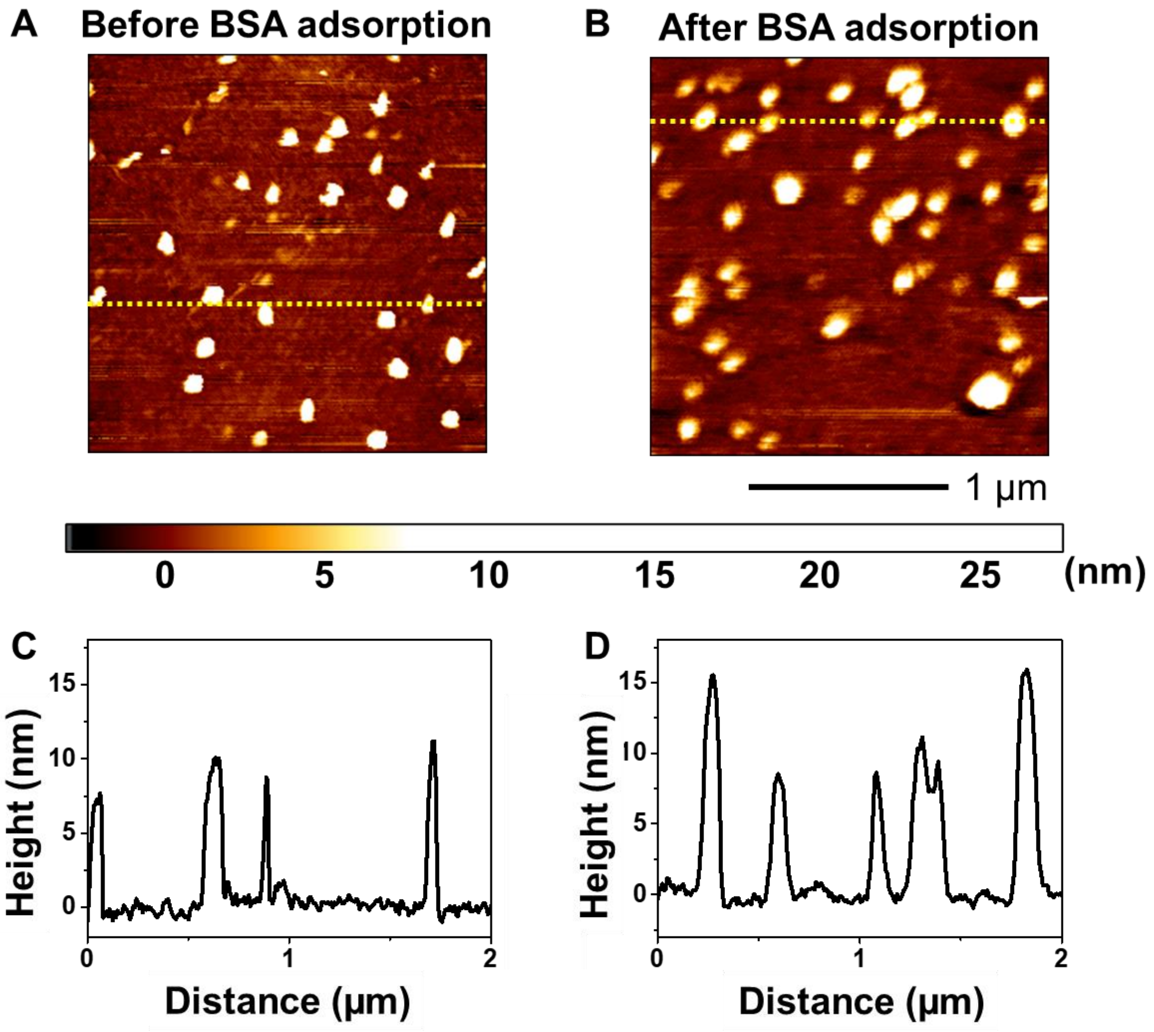

Figure S3. Surface morphology of AuNR platform **(A)** before and **(B)** after 0.01 µM BSA adsorption based on AFM measurements reported in height mode (scan area of 2 µm × 2 µm). **(C,D)** Corresponding line scan profiles of AuNR platforms and the results support protein attachment to the AuNR platform, as indicated by a ~5-nm increase in maximum height features that is consistent with the typical size of BSA protein molecules. The line scan profiles in panels (C) and (D) correspond to the dotted yellow lines in panels (A) and (B), respectively.

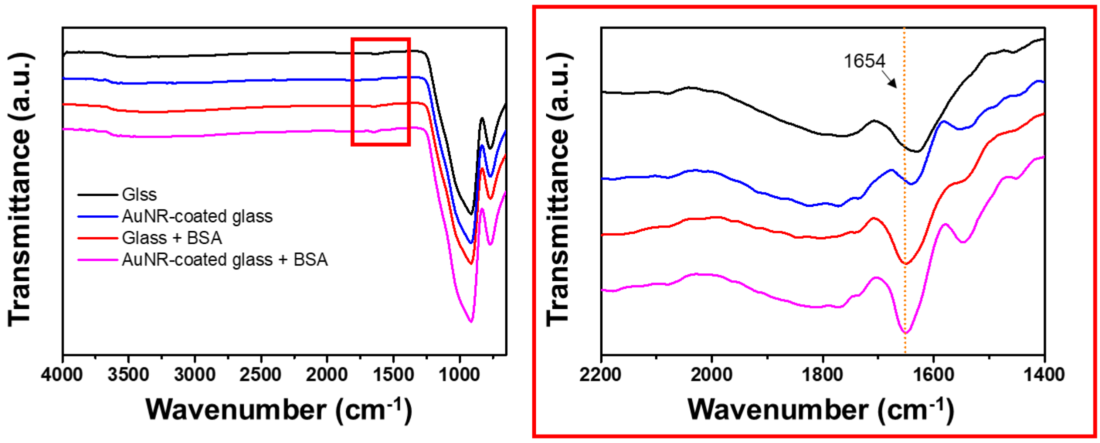

Figure S4. FTIR spectroscopic analysis of glass surface without and with BSA protein coating and AuNR-coated glass surface without and with BSA protein coating (scan range: 650–4000 cm^{-1}). Red box corresponds to a magnified view of the spectra (range: 1400 - 2200 cm^{-1}) and a peak at 1654 cm^{-1} is denoted that corresponds to the amide I band of attached BSA protein molecules. The BSA concentration was fixed at 100 μM for these experiments.

References

1. Bhardwaj, H.; Sumana, G.; Marquette, C.A. Gold nanobipyramids integrated ultrasensitive optical and electrochemical biosensor for Aflatoxin B1 detection *Talanta,* **2021**, *222*, 121578.
2. Trung, N.B.; Yoshikawa, H.; Tamiya, E.; Viet, P.H.; Takamura, Y.; Ashahi, T. Propitious immobilization of gold nanoparticles on poly (dimethylsiloxane) substrate for local surface plasmon resonance based biosensor. *Jpn. J. Appl. Phys.* **2012**, *51*, 037001.
3. Kyaw, H.H.; Al-Harthi, S.H.; Sellai, A.; Dutta, J. Self-organization of gold nanoparticles on silanated surfaces. *Beilstein J. Nanotechnol.* **2015**, *6*, 2345–2353.
4. Zhu, S.L.; Zhang, J.B.; Yue, L.Y.L.; Hartono, D.; Liu, A.Q. Label-free protein detection via gold nanoparticles and localized surface plasmon resonance. In Proceedings of the Adv. Mat. Res., 2009; pp. 95–98.
5. Calatayud-Sanchez, A.; Ortega-Gomez, A.; Barroso, J.; Zubia, J.; Benito-Lopez, F.; Villatoro, J.; Basabe-Desmonts, L. A method for the controllable fabrication of optical fiber-based localized surface plasmon resonance sensors. *Sci. Rep.* **2022**, *12*, 1–11.
6. Chen, H.; Zhao, L.; Chen, D.; Hu, W. Stabilization of gold nanoparticles on glass surface with polydopamine thin film for reliable LSPR sensing. *J. Colloid Interface Sci.* **2015**, *460*, 258–263.
7. Golmohammadi, S.; Etemadi, M. Analysis of plasmonic gold nanostar arrays with the optimum sers enhancement factor on the human skin tissue. *J. Appl. Spectrosc.* **2019**, *86*, 925–933.
8. Kooij, E.S.; Brouwer, E.M.; Wormeester, H.; Poelsema, B. Ionic strength mediated self-organization of gold nanocrystals: An AFM study. *Langmuir* **2002**, *18*, 7677–7682.
9. Kim, Y.-P.; Oh, E.; Hong, M.-Y.; Lee, D.; Han, M.-K.; Shon, H.K.; Moon, D.W.; Kim, H.-S.; Lee, T.G. Gold nanoparticle-enhanced secondary ion mass spectrometry imaging of peptides on self-assembled monolayers. *Anal. Chem.* **2006**, *78*, 1913–1920.
10. Nath, N.; Chilkoti, A. A colorimetric gold nanoparticle sensor to interrogate biomolecular interactions in real time on a surface. *Anal. Chem.* **2002**, *74*, 504–509.
11. Kumari, S.; Moirangthem, R.S. Portable and economical plasmonic capillary sensor for biomolecular detection. *Sens. Actuators, B* **2016**, *231*, 203–210.
12. Ferhan, A.R.; Hwang, Y.; Ibrahim, M.S.B.; Anand, S.; Kim, A.; Jackman, J.A.; Cho, N.-J. Ultrahigh surface sensitivity of deposited gold nanorod arrays for nanoplasmonic biosensing. *Appl. Mater. Today* **2021**, *23*, 101046.
13. Tunc, I.; Susapto, H.H. Label-free detection of ovarian cancer antigen CA125 by surface enhanced Raman scattering. *J. Nanosci. Nanotechnol.* **2020**, *20*, 1358–1365.
